# Acute Effect of Short Intensive Self-Myofascial Release on Jump Performance in Amateur Athletes: A Randomized Cross-Over Study

**DOI:** 10.3390/ijerph192416816

**Published:** 2022-12-14

**Authors:** Dawid Koźlenia, Jarosław Domaradzki

**Affiliations:** Unit of Biostructure, Faculty of Physical Education and Sport, Wroclaw University of Health and Sport Sciences, Al. I.J. Paderewskiego 35, 51-612 Wroclaw, Poland

**Keywords:** jump height, force, power, reactive strength index, foam rolling, young adults, athletes

## Abstract

Searching for effective methods to maximize physical performance that can be utilized during warm-ups is challenging in modern sports. This study aimed to investigate the effect of a short and intensive self-myofascial release (SI-SMR) on jumps in amateur, collegiate athletes. The study sample consists of 30 subjects with an average age of 21.8 years. The tests conducted included a squat jump (SJ), countermovement jump (CMJ), and drop jump (DJ). In the first week, half of the participants performed a standardized warm-up with additional short (15 s per lower limb muscle group) and intensive (20 reps/15 s) SMR and then performed jump tests. The other half performed a standard warm-up. The following week the groups switched interventions. The results revealed a tendency for all jump test parameters (height, force, and power), the reactive strength index, and stiffness to improve with SI-SMR, but the differences were small and insignificant. A dependent t-test for paired samples revealed that only SJ height improvement (+0.96 ± 2.63 cm) reached statistical significance (*p* = 0.04), but the small ES (ES = 0.14) could have attenuated this result. When a two-way mixed ANOVA was applied, the differences were insignificant. SI-SMR was ineffective in the direct improvement of jump performance. Although SI-SMR had no adverse effects, athletes should focus on specific preparations for sports competitions instead of using an SI-SMR protocol.

## 1. Introduction

Preparing for physical activity is crucial in professional and amateur sports. Therefore, coaches and athletes continue to search for a method to improve physical performance, even directly before a competition [1].

One of the best physical performance indicators is the vertical jump, which corresponds directly with force and power [2,3]. These parameters are reliable predictors of performance in many sports. Jump performance is related to anaerobic, mixed, and aerobic disciplines [4,5,6,7]. Moreover, jump test measurements can help predict injury risk [8]. Therefore, jump tests are considered a universal tool for measuring physical performance [9].

Manual therapies can help athletes during recovery and increase their physical performance [10,11]. Self-myofascial release (SMR) is a popular self-therapy associated with an improved range of motion and accelerated recovery [12]. The advantage of SMR is that the athlete can perform the procedure without the help of a therapist. Due to its simplicity and low cost, SMR has gained popularity among athletes of every level, and its effects on physical performance have been proven [13]. However, there is a lack of consensus on the optimal procedures to achieve the best results [14]. Even less is known about the acute effects of this procedure on jump performance [13]. The standard, longer procedure is ineffective in improving jump performance but has shown some benefits in the sprint [15]. However, a slow and long SMR enhances the range of motion [12]. Hughes and Ramer [14] postulated that improvements in mobility require at least 90 s of SMR. Conflicting studies have reported no impact and attenuated performance measures, while others have found improvement in performance scores [16,17,18,19].

Unfortunately, knowledge about the underlying mechanisms of the physiological response to SMR is still incomplete. Nevertheless, neurological, psychological, and physiological predispositions may influence the effects of SMR regardless of the procedure [13,20,21]. Thus highlighting the need to further explore the effects of SMR in various conditioning protocols.

The previous literature is limited to the use of SMR in direct preparation for physical effort. Especially, short protocols of SMR have not been fully researched. Therefore, the question of how a short and intensive SMR (SI-SMR) will affect performance remains. This study aimed to investigate the effects of SI-SMR on the lower limbs on the jump performance of amateur, collegiate athletes. To date, there are no studies investigating this type of SMR protocol on physical performance. Therefore, this study adds to the body of literature on SMR.

## 2. Materials and Methods

### 2.1. Participants

Before recruitment, a power calculation was conducted to determine the required sample size to detect a medium effect size (ES) using a paired samples *t*-test and a two-way mixed ANOVA [22]. To detect an ES > 0.6 with a power > 0.9 and an alpha value = 0.05, we need a minimum sample size of 26 subjects for the paired *t*-test and 30 subjects for the ANOVA. Both criterium were met.

Initially, 41 physical education students were recruited for this study. The inclusion criteria included no injury four weeks before the study, age 20–25 years old, being an active amateur athlete of one of the following disciplines requiring a high level of power: soccer, handball, basketball, volleyball, and extreme conditioning program training. Due to an injury before measurements (*n* = 1), rejection from the measurements (*n* = 1), not being active athletes (*n* = 5), and participating in extensive physical activity 48 h before the measurements (*n* = 4), 11 subjects were excluded. Finally, the study sample consists of 30 individuals (14 males and 16 females). A detailed description of the study sample is provided in Table 1.

All participants were volunteers and were required to sign a written consent before participating in this study. They were informed in detail about the purpose, type, research methodology, and participation conditions. Participants were allowed to withdraw from the research at any time without giving a reason. The participants were instructed to avoid extensive physical activity for 72 h before measurements, sleep for 8 h, and maintain their normal morning breakfast routine. An injury during or four weeks prior to the study excluded the participant from the study.

### 2.2. Intervention

This study was conducted in the Biokinetics Research Laboratory at the Wroclaw University of Health and Sport Sciences. The Quality Management System Certificate was PN-EN ISO 9001: 2009 (Certificate Reg. No. PW-48606-10E).

This study utilized a cross-over design (2 × 2) and was performed in a laboratory setting. The temperature in the room was 20 °C. There were three meetings between 7 a.m. and 11:30 a.m., separated by seven days when measurements were taken. During the first meeting, participants were familiarized with the methods and procedure. Somatic measurements were also performed. They were then randomly divided into two groups: A and B. The randomization was performed using the tool on the website www.randomization.com. A simple, non-returnable group randomization was performed. The following week, the jump measurements were performed. Group A performed a 10 min standard warm-up (consisting of 5 min of jogging, 15 reps of air squats, 15 reps of high knees, 15 reps of lunges, and submaximal trials of the jump test to be performed. The participants were allowed 3 to 5 trial jumps. Then, the SI-SMR was conducted prior to the jump test, whereas group B performed only a standard warm-up. SI-SMR was performed using a foam roller of 15 cm × 30 cm (Blackroll, Bottighofen, Switzerland). A tough foam roller was used to increase the stimulus. The participants were instructed to maintain high pressure, 7–8 on the pain numbering rating scale, [23] on the foam roller during application. The SI-SMR was performed on both lower limb muscle groups alternately, in the order of calves, hamstrings, glutes, and thighs. Each muscle group was targeted for 15 s with an intensity of 20 reps/15 s. The researchers supervised the participants to ensure proper technique and intensity using a metronome (Natural Metronome app, Single Minded Production, LLC, Margate, FL, USA). The participants used the metronome sound to indicate the tempo and viewed the time on the screen.

**Figure 1:**
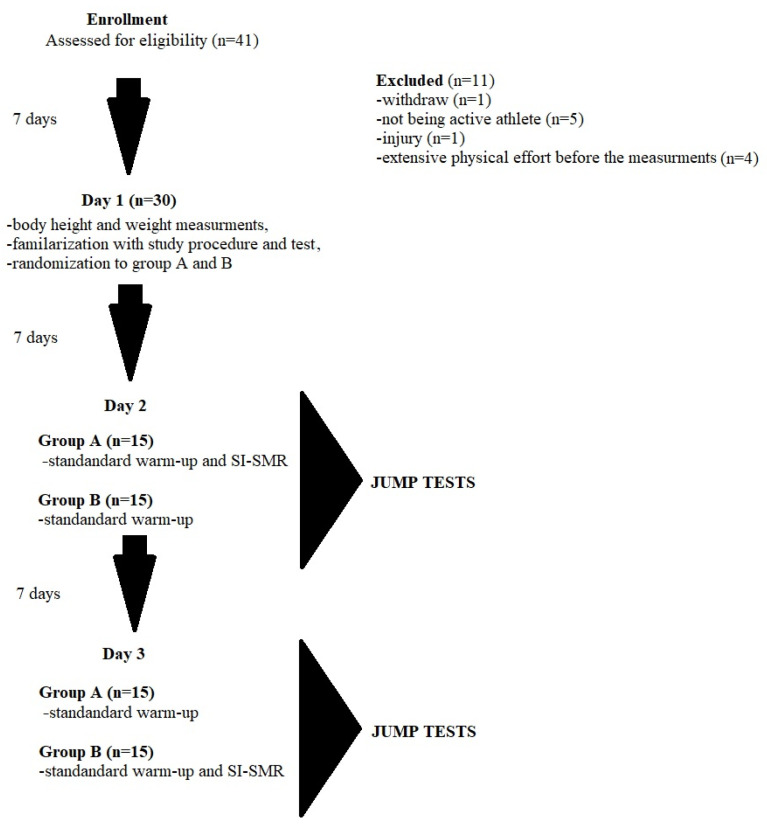
The study design.

Next meeting, the cross-over was performed—group A now performed only a standard warm-up, whereas group B performed the standard warm-up with the addition of SI-SMR. The jump tests were performed. The study design is presented in Figure 1.

### 2.3. Measurements

The height gauge model 764 (SECA, Hamburg, Germany) was used to measure body height and weight. Body mass index (BMI) was calculated based on the obtained results.

The parameters of the jump tests were measured using the scientifically validated mobile app for smartphones, MyJump2 [24,25]. Mobile apps have become popular in athletic testing due to the decreased cost without lowering reliability [26]. MyJump2’s validity and reliability have been confirmed [24,27,28]. Using measurements from MyJump2 in scientific studies is justifiable and has been used in experimental studies [29]. The iPhone version 13 (Apple Inc., Cupertino, CA, USA) was used. To calculate the jump parameters, a take-off frame and landing frame were manually selected from the video. The app then determines the jump height using the method described by Bosco et al. [30] with the jump height [m] = flight time^2^ [s] × 1.22625. All videos were taken and analyzed by the same evaluator with the same settings: videos were recorded from the frontal plane from a distance of 1.5 m with a standard calibration of 240 frames per second, as recommended in the manufacturer’s instructions.

Three jump tests were performed by each participant. Three attempts were performed for each jump test, separated by a 60 s break [8,31,32]. The tests were performed in random order. Participants were instructed to jump as high as possible. The best jump (jump height) was recorded and used in the analysis.

Squat jump (SJ)—the participant was instructed to flex their knees to 90° for 3 s and then jump vertically to their maximum height keeping their hands on their hips all the time. 

Countermovement Jump (CMJ)—the participant starts in a standing position with their hands on their hips. They were then instructed to make a fast downward movement (knee flexion to approximately 90°) and then make a quick upward movement jump as high as possible.

Drop Jump (DJ)—The participants maintained their hands on their hips for the entire jump. They dropped from a 40 cm box and were instructed to jump again as fast as possible to their maximal jump height keeping the landing phase as short as possible.

We analyzed jump height (cm), relative force, relative power, and eccentric utilization ratio (EUR) parameters, which were calculated based on the best CMJ height/SJ jump height [33]. This ratio provides insight into the stretch-shortening cycle (SSC). The DJ reactive strength index (RSI) was calculated using the following formula, jump height/contact time of the feet with the floor (milliseconds) [24], and stiffness [34] were also determined. All parameters were calculated automatically using the MyJump2 app [24,25].

### 2.4. Statistical Analyses

G*Power was used to perform statistical power and sample size calculations [35]. The Shapiro–Wilk test was performed to investigate the normality of the data, and the Levene test for the homogeneity of variance. Means, standard deviations, and confidence intervals (95%) were calculated. Cohen’s d values for ES were described as a value ≤ 0.2 equals a small ES, 0.5–0.79 equals a medium ES, and ≥0.8 equals a large ES [36]. To eliminate the bias of a period effect, unpaired t-tests were conducted concerning the time periods [37]. Paired *t*-tests for the dependent samples [38,39] and two-way mixed ANOVA (2 × 2) tests were used to compare the jump parameters before and after the SI-SMR protocol. The ES for the ANOVA test was calculated using eta-squared (η^2^, small = 0.01, moderate = 0.13, high = 0.26). The level of significance of this study was set at a *p*-value of <0.05. Statistica 13.0 (Statsoft Poland, Cracow, Poland) software was used for the analysis.

## 3. Results

In the first step of the analysis, the period effect was excluded by comparing the groups according to the time of the meetings. There were no statistical differences between groups from the two points in time for any parameter (*p* > 0.05). Next, a paired *t*-test was conducted to assess the effect of SI-SMR on the jump parameters (Table 2). The results revealed a tendency for SI-SMR to improve all jump test parameters. Both jump height, power, and force values improved in each jump test after the SI-SMR intervention. Moreover, the RSI and stiffness measurements during the DJ slightly improved. However, only in the case of SJ height, which had an improvement of 0.96 cm, was the difference statistically significant (*p* = 0.04). However, the small ES (ES = 0.14) attenuates this result. Furthermore, a visible improvement of 0.8 cm in DJ height was observed, but similar to other parameters, this change was insignificant (*p* = 0.10) with a small ES (ES = 0.12).

In the next step, the analysis was extended with a two-way mixed ANOVA. Again, the improvements in the jump parameters were still present, but there was a lack of statistical significance in all parameters tested (*p* > 0.05). The results of jump parameters for all the tests (pre- vs. post-intervention) are provided in Table 3.

## 4. Discussion

This study investigated the acute effect of SI-SMR on various jump parameters. There were no adverse effects noted in participants during the use of the SI-SMR protocol. Moreover, the tendency for improvement in many of the measured parameters was observed, with only SJ heights reaching statistical significance, but the differences were minor. However, this result is undermined; when ANOVA was performed, the statistical significance disappeared. Therefore, it can be speculated that using SI-SMR immediately before an activity seems ineffective due to the weak effect on performance.

SMR is used at every level of sporting activity as an effective tool in increasing flexibility or enhancing recovery [40]. Sulowska-Daszyk and Skiba [41] pointed out that SMR is useful in long-distance runners to improve flexibility and range of motion. These results were confirmed by Zhang et al. [42], who observed that the range of motion improved after SMR. They also reported improvements in dynamic balance and a significant effect on jump performance. These results are contrary to our observation. We did not find any improvement in jump parameters, but the SMR protocols between the two studies were different.

Similarly, Klich et al. [43] noted that the use of foam rolling did not result in any improvements in CMJ or sprints. However, their study did show an improvement in jump height after the soft tissue intervention (floss banding). One reason our study failed to show significant improvements may be the short intervention time despite the high intensity. However, prolonged duration of SMR may attenuate muscle power output [16], as well as endurance effort, which has been reported by Giovanelli et al. [17]. On the other hand, a short intervention of soft tissue stimulation (5–10 s) may bring about positive effects on the range of motion without electromyography (EMG) changes [44]. Similar results were noted by McDonald et al. [45], who noted an improved range of motion using SMR without an effect on EMG activity. Another study noted mobility improvements after SMR and suggested a possible mechanism for the improvements, in short, dynamic efforts after SMR use [16]. Additionally, Peacock et al. [19] observed improvements in power, strength, and agility after SMR. They used a short duration of SMR (30 s per body part), but in opposition to our study, they noted significant improvements in most of the measured parameters. Richman et al. [46] indicated that an SMR protocol consisting of 30 s per muscle group was a valuable tool during warm-ups compared to other methods. The previously mentioned study by Giovanelli et al. [17] performed myofascial release for 60 s per muscle group. This suggests that an SMR duration of less than 30 s per muscle group is too short to achieve significant effects. Sullivan et al. [43] suggested that the short duration of foam rolling was unable to appropriately stimulate the muscle groups to achieve any positive effects. These results raise the question of the optimal duration of SMR to produce an impact on specific motor abilities. When Godwin et al. [47] and Jones et al. [48] used 30 s of SMR per muscle group, they noted a small effect on jump parameters. However, prolongation of the SMR does not appear to be an effective solution [49]. Compared to our intervention, the abovementioned studies used a longer duration of therapy, which may be the main factor for its efficiency [17,19,43,44,46]. It is necessary to emphasize that most studies using various forms of SMR have not reported any adverse effects. However, some observations have reported SMR to have an attenuating effect on power and strength [16].

SMR cannot be unequivocally considered an effective method to enhance physical performance. The available studies vary in many aspects, such as the SMR protocol impedes their comparison and the ability to draw reliable conclusions [14]. Thus, further studies are warranted. Based on the current literature, athletes should focus on specific preparation before participating in their desired discipline [50].

A deeper insight into the biological mechanisms of the effects of SMR is needed. It has been proposed that SMR influences tissue stiffness and adhesion [20,51]. At the same time, it can alter nociceptor and mechanoreceptor sensitivities [21,52] and blood flow [20,51]. However, a psychological effect cannot be excluded. The associated feeling of improved well-being may produce a placebo effect [13,53].

Our study has some limitations. The athlete groups were heterogeneous and included athletes from various disciplines and sex. We did not use heart rate monitoring during the warm-up. We did not obtain baseline strength parameters of the lower limb muscles. On the other hand, the strength of this study is the novel approach to using an SI-SMR protocol. This is the first study to investigate the effectiveness of SI-SMR (15 s per muscle group) before physical effort.

Further studies are needed to evaluate if the improvements in jump performance would still be evident when various longevity breaks are introduced after SI-SMR and before the jump tests. It is also worth exploring the differences between participants who responded positively to SI-SMR (jump parameter improvements) and those who failed to benefit from an SI-SMR protocol.

## 5. Conclusions

SI-SMR appears to be ineffective when used immediately prior to physical effort. Although there are no adverse effects associated with SI-SMR, athletes should focus on sport-specific preparations for competitions instead of using an SI-SMR protocol directly before physical effort. There is a need to look for more effective interventions.

## Figures and Tables

**Table 1:** Characteristics of the study participants.

Group	General	Men	Women
Variable	Mean ± sd (95% CI)	Mean ± sd (95% CI)	Mean ± sd (95% CI)
Age (years)	21.8 ± 1.15 (21.36–22.23)	22.14 ± 1.41 (21.33–22.95)	21.50 ± 0.82 (21.06–21.94)
Body height (m)	1.74 ± 0.09 (1.71–1.78)	1.82 ± 0.08 (1.77–1.87)	1.69 ± 0.04 (1.671.71)
Body mass (kg)	70.06 ± 13.25 (65.11–75.01)	80.18 ± 11.83 (73.35–87.01)	61.22 ± 6.3 (57.86–64.57)
Body Mass Index (kg/m^2^)	22.72 ± 2.52 (21.78–23.66)	24.08 ± 2.16 (22.84–25.33)	21.54 ± 2.25 (20.34–22.74)
Training sessions per week (*n*)	3.76 ± 1.73 (3.11–4.41)	3.79 ± 1.63 (2.85–4.72)	3.75 ± 1.88 (2.75–4.75)
Single training session duration (min)	104.83 ± 26.01 (95.11–114.54)	98.21 ± 25.09 (83.73–112.70)	110.63 ± 26.20 (96.67–124.58)
Weekly training volume (hours/week)	6.48 ± 3.12 (5.31–7.64)	6.28 ± 3.23 (4.42–8.14)	6.66 ± 3.12 (4.99–8.32)
Sport experience (years)	8.9 ± 3.79 (7.48–10.31)	8.36 ± 4.29 (5.88–10.83)	8.06 ± 4.04 (5.91–10.22)

**Table 2:** Paired t-test results between jump parameters pre- (No SI-SMR) and post-intervention (SI-SMR).

Test (Jump)	Variable	Mean ± SD 95% CI	Mean ± SD 95% CI	Mean ± SD 95% CI	Effect Size	t	*p*
		NO SI-SMR	SI-SMR	Difference			
Squat Jump (SJ)	Jump height [cm]	27.39 ± 6.46 25.10–29.69	28.35 ± 6.86 25.91–30.79	0.96 ± 2.63 0.02–1.89	0.14	−2.09	0.0443 *
	Relative force [N/kg]	18.30 ± 1.84 17.65–18.95	18.50 ± 2.43 17.64–19.36	0.20 ± 1.84 −0.45–0.85	0.09	−0.63	0.5321
	Relative power [W/kg]	21.29 ± 4.47 19.70–22.87	21.92 ± 5.33 20.03–23.81	0.63 ± 2.84 −0.37–1.64	0.13	−1.28	0.2091
Counteromovemnt Jump (CMJ)	Jump height [cm]	28.21 ± 6.67 25.84–30.57	28.59 ± 6.61 26.25–30.94	0.39 ± 1.80 −0.25–1.03	0.06	−1.24	0.2243
	Relative force [N/kg]	18.57 ± 1.99 17.86–19.27	18.56 ± 2.27 17.75–19.36	0.00 ± 1.75 17.76–19.37	0.00	0.01	0.9911
	Relative power [W/kg]	21.93 ± 4.78 20.23–23.62	22.06 ± 4.95 20.30–23.82	0.13 ± 2.35 −0.70–0.96	0.03	−0.31	0.7569
CMJ height/ SJ height	Eccentric Utilization Ratio	1.03 ± 0.09 1.00–1.06	1.01 ± 0.07 0.98–1.03	0.02 ± 0.09 0.01–0.05	0.02	1.23	0.2273
Drop Jump (DJ)	Jump height [cm]	28.32 ± 6.62 25.97–30.67	29.11 ± 6.05 26.9731.26	0.80 ± 2.72 −0.017–1.76	0.12	−1.69	0.1017
	Relative force [N/kg]	18.65 ± 2.13 17.89–19.40	18.75 ± 2.22 17.97–19.54	0.11 ± 1.97 −0.59–0.80	0.05	−0.31	0.7605
	Relative power [W/kg]	22.06 ± 4.95 20.31–23.82	22.48 ± 4.71 20.81–24.15	0.42 ± 3.08 −0.67–1.51	0.08	−0.77	0.4445
	Reactive Strength Index	1.46 ± 0.41 1.31–1.60	1.52 ± 0.52 1.33–1.70	0.06 ± 0.34 −0.05–0.18	0.13	−1.05	0.3026
	Stiffness [kN/m]	10.36 ± 4.32 8.82–11.89	11.06 ± 6.85 8.64–13.49	0.71 ± 5.35 −2.60–1.19	0.12	−0.76	0.4535

* Statistically significant *p* < 0.05.

**Table 3:** Pre-to post-intervention changes in jump parameters results of a two-way repeated measures ANOVA.

Jump Test	Parameters	Group	NO SI-SMR	SI-SMR	Differences	F	*p*	η^2^
			Mean ± SD 95% CI	Mean ± SD 95% CI				
Squat Jump (SJ)	Jump height [cm]	A	27.04 ± 1.77 (23.48–30.61)	28.74 ± 1.77 (25.18–32.30)	1.70	0.004	0.9490	0.01
		B	27.72 ± 1.90 (23.91–31.53)	27.83 ± 1.90 (24.02–31.63)	0.11			
	Relative force [N/kg]	A	18.40 ± 0.56 (17.26–19.54)	18.90 ± 0.56 (17.76–20.04)	0.5	0.904	0.3459	0.01
		B	18.02 ± 0.60 (16.80–19.24)	18.16 ± 0.60 (16.94–19.38)	0.14			
	Relative power [W/kg]	A	21.25 ± 1.30 (18.63–23.87)	22.57 ± 1.30 (19.95–25.19)	1.32	0.268	0.6066	0.01
		B	21.13 ± 1.39 (18.33–23.93)	21.29 ± 1.39 (18.49–24.09)	0.16			
Counter-movemnt Jump (CMJ)	Jump height [cm]	A	27.96 ± 1.77 (24.41–31.51)	28.85 ± 1.77 (25.30–32.40)	0.89	0.002	0.9602	0.001
		B	28.41 ± 1.89 (24.61–32.20)	28.23 ± 1.89 (24.43–32.02)	−0.18			
	Relative force [N/kg]	A	18.67 ± 0.53 (17.54–19.80)	18.90 ± 0.56 (17.77–20.03)	0.23	0.724	0.3984	0.01
		B	18.15 ± 0.60 (16.94–19.37)	18.42 ± 0.60 (17.21–19.63)	0.27			
	Relative power [W/kg]	A	21.96 ± 1.29 (19.36–24.56)	22.57 ± 1.29 (19.97–25.16)	0.61	0.213	0.6461	0.01
		B	21.44 ± 1.38 (18.66–24.22)	21.85 ± 1.38 (19.07–24.63)	0.41			
CMJ height/ SJ height	Eccentric Utilization Ratio	A	1.02 ± 0.02 (0.98–1.06)	1.03 ± 0.02 (0.99–1.07)	0.01	0.598	0.4427	0.01
		B	1.01 ± 0.02 (0.97–1.05)	1.04 ± 0.02 (1–1.09)	0.03			
Drop Jump (DJ)	Jump height [cm]	A	28.03 ± 1.69 (24.64–31.43)	29.14 ± 1.69 (25.75–32.53)	1.11	0.014	0.9078	0.01
		B	28.56 ± 1.81 (24.93–32.19)	29.02 ± 1.81 (25.39–32.65)	0.46			
	Relative force [N/kg]	A	18.71 ± 0.57 (17.56–19.87)	19.03 ± 0.57 (17.87–20.18)	0.32	0.401	0.5293	0.01
		B	18.43 ± 0.61 (17.19–19.67)	18.55 ± 0.61 (17.32–19.79)	0.12			
	Relative power [W/kg]	A	22.06 ± 1.28 (19.47–24.64)	22.85 ± 1.28 (20.27–25.43)	0.79	0.104	0.7486	0.01
		B	22.02 ± 1.37 (19.21–24.92)	22.12 ± 1.39 (19.26–24.78)	0.10			
	Reactive Strength Index	A	1.43 ± 0.12 (1.18–1.67)	1.40 ± 0.12 (1.16–1.65)	−0.03	1.235	0.2710	0.02
		B	1.48 ± 0.13 (1.21–1.74)	1.64 ± 0.13 (1.37–1.90)	0.16			
	Stiffness [kN/m]	A	9.9 ± 1.48 (6.93–12.86)	13.2 ± 1.58 (10.03–16.38)	3.31	2.67	0.1075	0.04
		B	9.14 ± 1.48 (6.18–12.11)	10.85 ± 1.58 (7.68–14.03)	−1.71			

## Data Availability

The raw data supporting the conclusions of this article will be made available by the authors without undue reservation.
